# Healthy Skin

**Published:** 1877-05

**Authors:** 


*Healthy Skin; a Popular Treatise on the Skin and Hair. Their Preservation and Management.* By ERASMUS WILSON, F. R. S., F. R. C. S., Professor of Dermatology in the Royal College of Surgeons of England. Eighth edition. Philadelphia: Lindsay & Blakiston, 1876.

Of a book that has already passed to its eighth edition little need be said. But if people purchase this book thinking to learn from it the entire management of the skin in disease as well as in health, they will find that they have mistaken its scope.

In the preface to this edition the author indicates his belief that it might have been made better, and we agree with him.

A beautiful skin and abundant hair are not by any means invariable indices of good health, and certain defects in them often detract very little, or not at all, from vigor of health, or usefulness of life. With beauty or deformity, agreeableness or disagreeableness of appearance, this subject has much to do. Of the treatment of diseases of the skin it would probably be as well to say nothing as to give the very meagre instruction that is here given. That part which relates to the microscopical anatomy of the skin and hair is excellent but nothing is added to what is already very generally known of the influence of diet, exercise and clothing.

With regard to ablutions, there is more occasion to find fault with the author for leaving important things unsaid, than for saying what he does. Of the two things, frequent change of underclothing is no doubt more important than frequent ablutions of the covered portions of the body, especially for children. Many children bear bathing very poorly, particularly during the process of teething, or when the health is at all disturbed. The same may be said of invalids, and people of impaired vitality. To inculcate the use of a daily bath, almost as being the chief end, or means of existence, is mischievously overdoing the matter. It is not safe ground; and should be sharply hedged about with restrictions. Healthy young babies enjoy a daily bath, agreeably and quickly given, in a warm room, and a corresponding change of linen, up to the fifth, sixth, or seventh month, according as teething is early or late. But that once well commenced, it is better to wash only the more exposed and more soiled portions of the body several times daily, keep the child in soft, clean, delicate, woolen underclothing, thick or thin, according to the season, and make the entire bath only a weekly instead of a daily operation. Although the anatomy of the skin is now very well understood its functions, as an entire organ, can still be studied with advantage. We do not know all about it, and while no one can for a moment doubt the expediency or necessity of a weekly or semi-weekly bath taken so as not to chill or overweary the bather, or interfere with digestion, those books which teach the use of a daily bath, save for exceptional cases of full health and vigor and maturity of life, are calculated to do harm. Even a horse may be injured by too much washing; how much more a human being, of superior delicacy and susceptibility of nervous organization, who must disrobe, and be subjected to considerable change of temperature without the accompanying stimulus to the respiratory processes which the open air gives. Public bathing houses, and houses for the washing of clothing, are a necessity to the poor, and should be kept as a charity in every city, as much as the public drinking fountain, and the winter soup house. But to the children of refinement and luxury, to children of decayed or decaying families, to people in delicate or failing health, and to those past the meridian of life, we would say, use plenty of good food, and clean, entirely comfortable clothing, take plenty of fresh air, in an agreeable way, but beware of excessive bathing. And this might be said, as well, to those who follow fatiguing, exhausting employments. With these restrictions, the daily bath becomes a necessity only in exceptional cases. Of it as a luxury we have nothing to add to what the author says of it, except, that like many other luxuries, it may be, and is debilitating. That diseased conditions of the skin are often prejudicial and destructive to good health no one would think of denying, but these popular medical treatises must teach a great deal very briefly, very clearly, and very carefully, or they are more mischievous than useful; and this neither teaches enough hygiene, nor enough medicine, to fulfil its object. A really good popular treatise on this subject would be one that would add to the anatomy here given all that is really and absolutely known about the care of the skin in health and disease, and nothing which is not definitely and forever settled.

